# Identification of a novel compound heterozygous mutation and a homozygous mutation of *SLURP1* in Chinese families with Mal de Meleda

**DOI:** 10.1186/s12920-023-01580-1

**Published:** 2023-07-01

**Authors:** Tian Wang, Zhuangli Tang, Tong Xiao, Junru Ren, Shuyao He, Yan Liu, Shengxiang Xiao, Xiaopeng Wang

**Affiliations:** 1 Department of Dermatology, The Second Affiliated Hospital of Xi’an Jiaotong University, 157 Xiwu Road, Xi’an, 710004 China; 2 Department of Dermatology, The Second Affiliated Hospital of Zhejiang University, Hangzhou, China

**Keywords:** Mal de Meleda, *SLURP1*, *Mutation analysis*

## Abstract

**Background:**

Mal de Meleda is an autosomal recessive palmoplantar keratoderma, with *SLURP1* identified as the pathogenic gene responsible. Although over 20 mutations in *SLURP1* have been reported, only the mutation c.256G > A (p.G87R) has been detected in Chinese patients. Here, we report a novel heterozygous *SLURP1* mutation in a Chinese family.

**Methods:**

We assessed the clinical manifestations of two Chinese patients with Mal de Meleda and collected specimens from the patients and other family members for whole-exome and Sanger sequencing. We used algorithms (MutationTaster, SIFT, PolyPhen-2, PROVEAN, PANTHER, FATHMM, mCSM, SDM and DUET) to predict the pathogenetic potential of the mutation detected. We also employed AlphaFold2 and PyMOL for protein structure analysis.

**Results:**

Both patients displayed the typical manifestation of palmoplantar keratoderma. In Proband 1, we detected a novel compound heterozygous mutation (c.243C > A and c.256G > A) in exon 3 of *SLURP1*. Proband 2 was an adult female born to a consanguineous family and carried a homozygous mutation (c.211C > T). Algorithms indicated both mutations to be probably disease causing. We used AlphaFold2 to predict the protein structure of these mutations and found that they cause instability, as shown by PyMOL.

**Conclusions:**

Our study identified a novel compound heterozygous mutation (c.243C > A and c.256G > A) in a Chinese patient with Mal de Meleda that has the potential to cause instability in protein structure. Moreover, this study expands on the existing knowledge of *SLURP1* mutations and contributes to knowledge of Mal de Meleda.

**Supplementary Information:**

The online version contains supplementary material available at 10.1186/s12920-023-01580-1.

## Background

Mal de Meleda (MdM; OMIM# 248300), also known as keratosis palmoplantaris transgrediens of Siemens, is an autosomal recessive genodermatosis [1]. MdM is a type of palmoplantar keratoderma (PPK) that was first described by the physician Luca Stulli from Dubrovnik on the Adriatic Island of MIjet (Meleda) in 1826 [2]. The estimated prevalence of MdM in the general population is 1/100,000 [3]. MdM is characterized by sharply demarcated erythema and hyperkeratosis of the palms and soles beginning in infancy and progressing with age (progrediens) [4], with the lesions gradually extending to the dorsal aspects of the hands and feet (transgrediens). Histological features of MdM include hyperkeratosis, hypergranulosis, and acanthosis without epidermolysis, accompanied by mild to moderate perivascular lymphocytic infiltration of the dermis [5].

SLURP1 (secreted lymphocyte antigen 6/urokinase-type plasminogen activator receptor-related protein 1) belongs to the Ly-6/uPAR superfamily, characterized by a three-finger folded structure in a snake toxin-like form [6]. SLURP 1 is encoded by the *ARS component B (ARS B)* gene (now known as the *SLURP1* gene), which is located on the long arm of chromosome 8q24-qter. It is reported that SLURP1 promotes keratinocyte apoptosis while inhibiting proliferation of keratinocytes [7]. SLURP1 also upregulates expression of differentiation markers including transglutaminase 1(TGM1) and cytokeratin 10 (CK10) in keratinocytes [8].

Defects in the gene encoding SLURP1 have been reported to cause MdM [9]. Indeed, mutations in *SLURP1* have been proven to affect the expression and integrity of the SLURP1 protein in MdM patients [10], and Slurp1 deficient mice show severe PPK phenotypes owning to keratinocyte proliferation [11]. Additionally, approximately 20 *SLURP1* gene mutations have been examined by biochemical analysis in vitro[12]. Although mutations in *SLURP1* have been proven to be involved in the pathogenesis of MdM, the functions and structures of some mutations remain unclear, and knowledge of variants is limited. The advent of AlphaFold2 constitutes dramatic progress in bioinformatic analysis and structural biology, showing unprecedented levels of accuracy in modelling single-chain protein structures [13].

This study included two Chinese families with MdM. The clinical phenotypes were recorded. The *SLURP1* gene was sequenced in the patients and their families for diagnosis. A novel compound heterozygous mutation in exon 3 was detected. Then, we analyzed the pathogenicity of this novel mutation by using mutation prediction software. We then applied AlphaFold2 to predict the effects on structure of the mutations and analyze the resulting changes in protein structure. We also review the current knowledge on *SLURP1* mutations associated with MdM.

## Methods

### Study subjects

Two patients from different Chinese families were examined at the Second Affiliated Hospital of Xi’an Jiaotong University. Both patients were diagnosed clinically with PPK and treated for several years. The patients and their parents agreed to be examined clinically and to provide blood samples for genetic analysis. The research described in this manuscript was approved by the Ethical Committee of the Second Affiliated Hospital of Xi’an Jiaotong University. Informed consent was provided by both patients and their families.

### Gene sequencing and analysis

A dermatologic specialist performed the clinical assessment and recorded the family background. Peripheral blood samples were collected from the patients and their families. A total of 100 healthy individuals randomly selected from medical staff and from medical students at our institution were used as healthy controls to assess gene polymorphisms.

Whole-exome sequencing and Sanger sequencing were conducted. Extraction of genomic DNA was performed according to the manufacturer’s protocol. Primers flanking all 3 exons and the adjacent introns of the *SLURP1* gene were designed. Polymerase chain reaction (PCR) of the *SLURP1* gene was performed using allele-specific primers following standard conditions, and the products were sequenced by Kangso Medical Inspection. Novel mutations were identified by comparing the sequences with previously published articles and currently available databases, including HGMD Pro, PubMed, and dbSNP.

### Mutation analysis and protein structure prediction

MutationTaster (http://www.mutationtaster.org/) was utilized to predict pathogenicity of the gene mutations identified. The mutation cut-off scanning matrix (mCSM) method (http://biosig.unimelb.edu.au/mcsm/) and Site Directed Mutator (SDM; http://marid.bioc.cam.ac.uk/sdm2) were used to evaluate free energy changes. The DUET server (http://biosig.unimelb.edu.au/duet/) was employed to assess changes in stability caused by the mutations. SIFT (http://sift.jcvi.org/) and Polyphen2 (http://genetics.bwh.harvard.edu/pph2/) were used to predict SNVs and INDELS.

We applied AlphaFold2 (https://colab.research.google.com/github/sokrypton/ColabFold/blob/main/AlphaFold2.ipynb) to elucidate the molecular structure of SLURP1 and perform comparative modelling. Changes in chemical bonds after missense mutation was assessed using the molecular visualization tool PyMOL.

## Results

### Examination of cutaneous manifestations

**Fig. 1:**
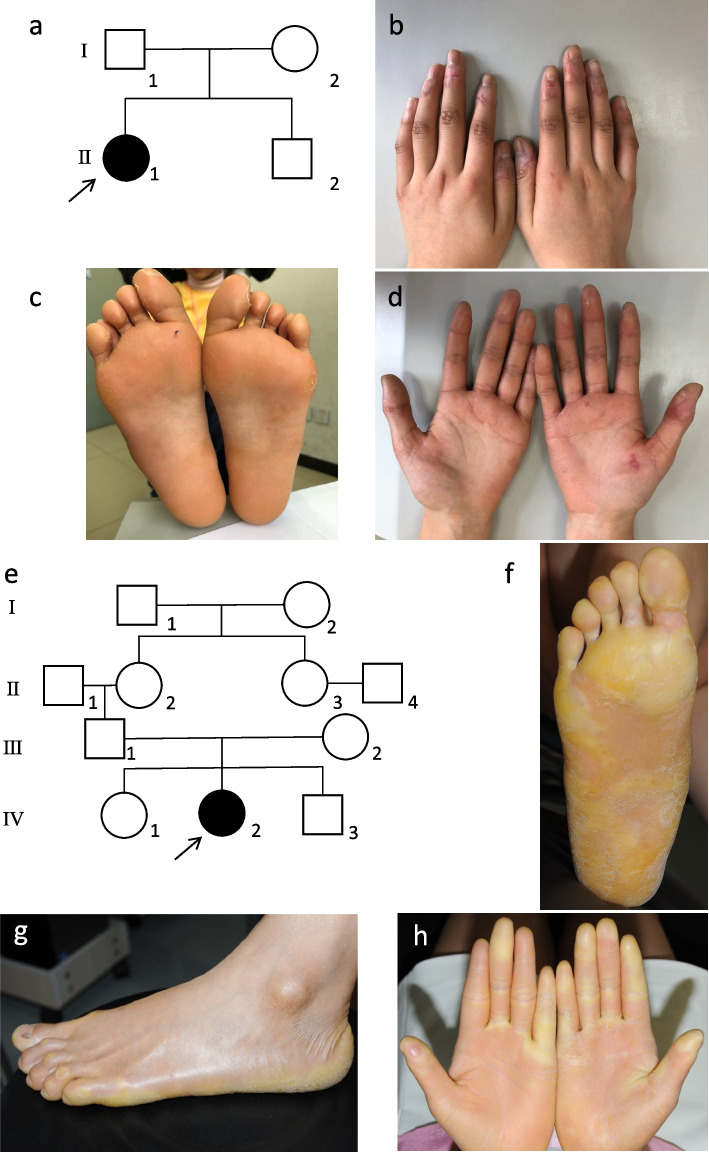
Clinical manifestations and pedigree of patients. **a** Pedigree of proband 1. Arrow represents the proband, square represents male, and circle represents female. **b** Palmoplantar keratoderma in proband 1 (P1). **c-d** Mild hyperkeratotic and erythematous plaques involve the palms and soles with scales. **e** Pedigree of proband 2, who was the only affected individual in a consanguineous family. **f-h** Yellow keratoderma and erythema involve dorsa of the hands and feet. Hyperhidrosis and maceration appear on the palms and soles

**Fig. 2:**
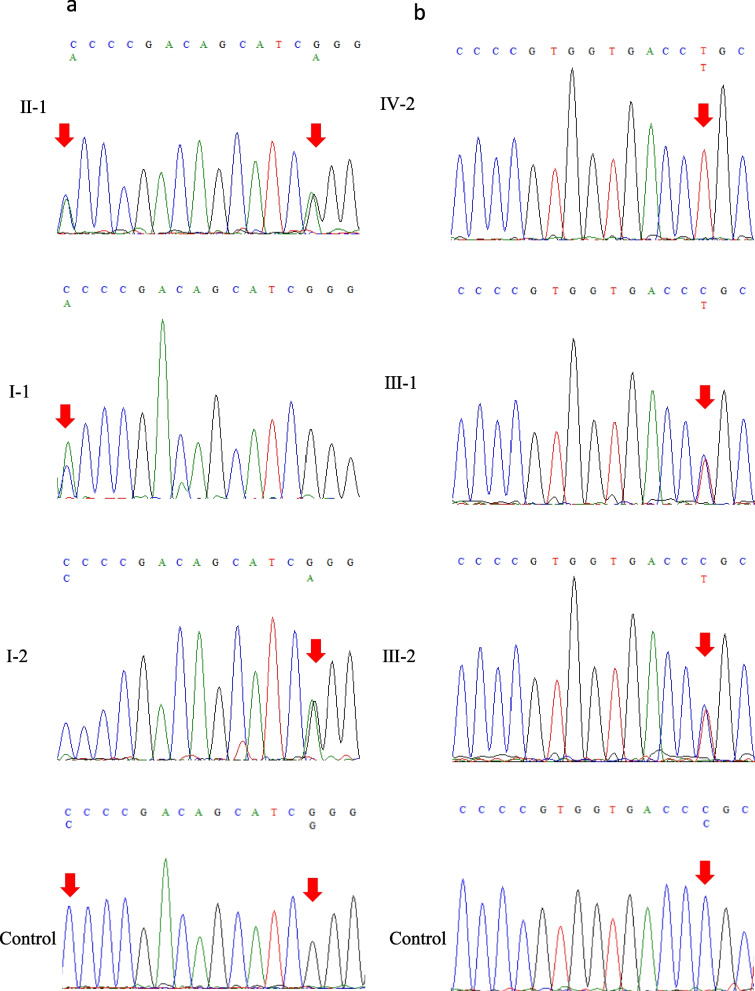
Missense mutations of *SLURP1*. **a** P1(II-1): a compound heterozygous mutation c.243C > A, c.256G > A. P1 father (I-1): a heterozygous mutation c.243C > A. P1 mother (I-2): a heterozygous mutation c.256G > A. **b** P2 (IV-2): a homozygous mutation c.211C > T. P2 father (III-1): a heterozygous mutation c.211C > T. P2 mother (III-2): a heterozygous mutation c.211C > T. Normal human control for mutations were shown

Proband 1 (P1) (Fig. 1a, II-1) was an 11-year-old Chinese female from a family with no known consanguinity. She developed hyperkeratotic plaques on her palms and soles at the age of 5 years. Physical examination showed that the lesions were restricted to the palms and soles, without extending to the upper arms or legs (Fig. 1b-d). No involvement of the fingernails, toenails or mucosa was observed. The lesions were seldom painful or itchy. The patient did not have malodorous fissures or macerations. Her parents (Fig. 1a, I-1,2) did not have such symptoms.

Proband 2 (P2) (Fig. 1e, IV-2) was a 27-year-old female from a consanguineous family. She visited our department complaining of PPK on her palms and soles, with repeated infections. Erythema and blisters appeared on her heel at the age of 1 year. The lesions gradually extended to her upper arms and legs, accompanied by painful fissures and macerations, which could be aggravated by trauma. Physical examination revealed diffuse erythema with thick, waxy, yellow scales on her palms and soles, extending to the dorsal aspects of the hands and feet (Fig. 1f-h). Some of her nails were also affected.

### Identification of *SLURP1* gene mutations

As the two probands both declared that lesions developed on their extremities at young ages and progressed with time, we decided to perform gene testing to determine whether the condition was caused by gene mutations. Specimens were collected from the probands and their parents. PCR products were analyzed by Sanger sequencing. The results revealed different pathogenic mutations in these two patients. A compound heterozygous mutation, c.243C>A and c.256G>A (Figure 2a, II-1), was detected in exon 3 of the *SLURP1* gene in Proband 1 (P1). Her parents were both heterozygous carriers with no clinical symptoms. Her father carried c.243C>A (Figure 2a, I-1), and her mother carried the heterozygous c.256G>A mutation (Figure 2a, I-2). We screened the mutations in Database of Single Nucleotide Polymorphisms (dbSNP), Human Gene Mutation Database (HMGD), Exome Aggregation Consortium (ExAC), Genome Aggregation Database (gnomAD) and 1000 Genomes to confirm that c.243>A is a novel mutation. Based on the results of whole-exome sequencing, Proband 2 (P2) carried the homozygous mutation c.211C>T (Figure 2b, IV-2). With further investigation, we found that her parents carried heterozygous mutations without manifestations (Figure 2b, III-1,2). Neither of these two mutations were found in 100 healthy people (Figure 2b).

### Prediction of protein structure and mutation impacts

**Fig. 3:**
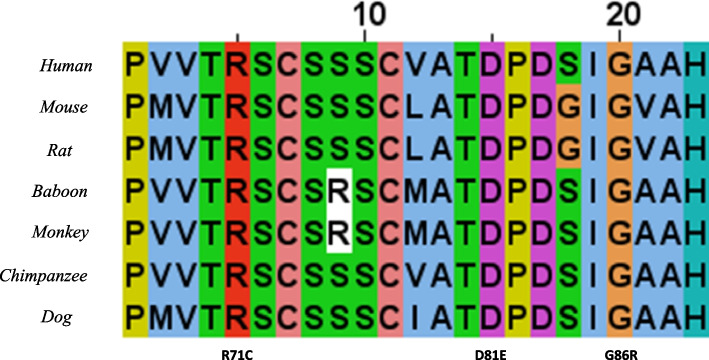
Conservation of the local amino acid sequence by multiple alignment of SLURP1 from different species. R71C, D81E and G86R are highly conservative positions

We searched “SLURP1” in the UniProt database and selected sequences from species on the list to perform multiple alignment of these sequences. The results showed that the amino acids at residues 71, 81 and 86 are highly conserved (Fig. 3). According to MutationTaster, the mutation c.243C > A(Asp81Glu) is predicted to be a polymorphism, whereas c.256G > A(Gly86Arg) is predicted to be disease causing. SIFT predicts c.243C > A as damaging c.256G > A as tolerated. PolyPhen-2 and Provean indicate that these two mutations are probably damaging and deleterious. The impact of the c.211C > T mutation is predicted to be damaging by all algorithms (Table 1).

**Table 1:** *SLURP1* exome mutations: review and in silico predictions for functions

Nucleotide change	Protein change	Mutation taster		PROVEAN	SIFT		PolyPhen-2		PANTHER		FATHMM		mCSM	Site Directed Mutator	DUET
c.1A> C	M1L	0.0001	disease causing	-0.76	0.000	damaging	0.267	benign	97	probably benign	-1.52	damaging	0.038 kcal/mol (Stabilizing)	0.0 (Reduced stability)	0.497 kcal/mol (Stabilizing)
c.43 T > C	W15R	0.0000	disease causing	-1.79	0.139	tolerated	0.995	probably damaging	97	probably benign	-1.59	damaging	0.057 kcal/mol (Stabilizing)	-0.14 (Reduced stability)	0.04 kcal/mol (Stabilizing)
c.129C > A	C43X	1.0000	disease causing												
c.211C > T*	R71C	0.9446	Deleterious	-5.4	0.001	damaging	0.895	possibly damaging	98	probably benign	-0.85	tolerated	-1.722 kcal/mol (Destabilizing)	-1.45 (Reduced stability)	-1.995 kcal/mol (Destabilizing)
c.212G > A	R71H	0.9565	polymorphism	-3.32	0.007	damaging	0.998	probably damaging	98	probably benign	-0.83	tolerated	-0.647 kcal/mol(Destabilizing)	-0.75 (Reduced stability)	-0.722 kcal/mol (Destabilizing)
c.212G > C	R71P	0.8811	polymorphism	-3.98	0.006	damaging	0.988	probably damaging	98	probably benign	-0.83	tolerated	-0.628 kcal/mol (Destabilizing)	-2.69 (Reduced stability)	-1.321 kcal/mol (Destabilizing)
c.229 T > C	C77R	0.9009	disease causing	-11.01	0.000	damaging	1	probably damaging	98	probably benign	-1.12	tolerated	-0.05 kcal/mol (Destabilizing)	-0.72 (Reduced stability)	0.151 kcal/mol (Stabilizing)
c.243C > A*	D81E	0.7546	polymorphism	-2.52	0.013	damaging	0.925	probably damaging	98	probably benign	-0.49	tolerated	-0.212 kcal/mol (Destabilizing)	-0.49 (Reduced stability)	-0.075 kcal/mol (Destabilizing)
c.244C > T	P82S	0.6831	polymorphism	-1.61	0.053	tolerated	1	probably damaging	98	probably benign	-0.26	tolerated	-0.343 kcal/mol (Destabilizing)	0.1 (Increased stability)	-0.164 kcal/mol (Destabilizing)
c.256G > C	G86R	0.0035	disease causing	-5.48	0.095	tolerated	1	probably damaging	98	probably benign	-0.56	tolerated	-0.449 kcal/mol (Destabilizing)	-3.82 (Reduced stability)	-0.842 kcal/mol (Destabilizing)
c.256G > A*	G86R	0.0035	disease causing	-5.48	0.095	tolerated	1	probably damaging	98	probably benign	-0.56	tolerated	-0.449 kcal/mol (Destabilizing)	-3.82 (Reduced stability)	-0.842 kcal/mol (Destabilizing)
c.280 T > A	C94S	0.9847	disease causing	-9.300	0.000	damaging	0.999	probably damaging	1036	probably damaging	-5.5	damaging	-1.535 kcal/mol (Destabilizing)	-0.63 (Reduced stability)	-1.306 kcal/mol (Destabilizing)
c.286C > T	R96X	0.9641	disease causing												
c.293 T > C	L98P	1.0000	disease causing	-6.7	0.000	damaging	1	probably damaging	324	possibly damaging	-0.87	tolerated	-1.422 kcal/mol (Destabilizing)	-1.87 (Reduced stability)	-1.831 kcal/mol (Destabilizing)
c.296G > A	C99Y	0.9935	disease causing	-10.3	0.001	damaging	1	probably damaging	1036	probably damaging	-6.25	damaging	-0.898 kcal/mol (Destabilizing)	0.93 (Increased stability)	-0.54 kcal/mol (Destabilizing)

MutationTaster 0–1 (disease causing, disease causing automatic, polymorphism, polymorphism automatic),SIFT < 0.50 damaging; > 0.50 tolerated, PolyPhen-2 0–1 (< 0.447 neutral; 0.447 ≤ possible damaging ≤ 0.909; probably damaging > 0.909), PROVEAN > 1.3: neutral; < 4.1: deleterious; 1.3 to 2.5: possibly neutral; 2.5 to 4.1: possibly deleterious. PANTHER time > 450 my, probably damaging; 450 my > time > 250 my, possibly damaging; time < 250 my, probably benign (“my’’ is an abbreviation of ‘‘millions of years’’), FATHMM < -1.5 damaging; ≥ 1.5 tolerated. mCSM, Site Directed Mutator and DUET > 0 increase stability; < 0 reduce stability

**Fig. 4:**
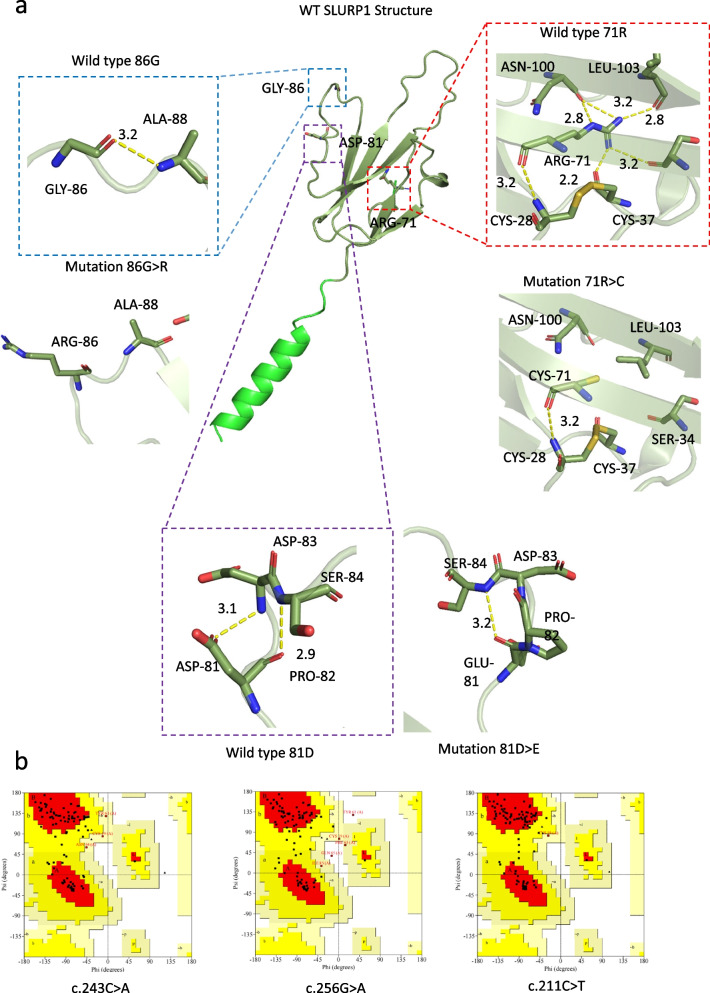
The impacts of *SLURP1* mutations on the molecular structure of protein. **a** AlphaFold2 predicted the wild type and mutated protein crystal structure (domain in smudge). The hydrogen bonds were shown by yellow lines. The unit of the distances was Å. **b** Ramachandran plots of mutated proteins were provided

We also used AlphaFold2 to assess the protein crystal structure of SLURP1, with significant structural changes found between the wild-type and mutated proteins (Fig. 4a, b). In the wild-type SLURP1 protein, Asp-81 forms two hydrogen bonds with Asp-83 and Ser-84, at 3.1 Å and 2.9 Å, respectively. However, D81E decreases hydrogen bonding with Asp-83 and results in reforming of a 3.2 Å hydrogen bond with Ser-84. The mutation c.256G > A would result in no hydrogen bonds between Gly-86 and Ala-88. R71 forms many hydrogen bonds with spatially nearby amino acids, but R71C only maintains one hydrogen bond with Cys-28. All these predictions indicate that both the compound heterozygous mutation and homozygous mutation result in unstable protein structures, which coincide with the predictions by mCSM, SDM and DUET (Table 1).

## Discussion

MdM is a rare inherited subtype of PPK. It was first reported by Luca Stulli but redefined by Hovorka and Ehlers as MdM in 1897, and this definition has been used until now [14]. Hereditary PPK diseases can be divided into five basic categories: diffuse PPK, diffuse mutilating PPK, focal PPK, ectodermal dysplasia with PPK, and syndromic PPK [4] and Nagashima-type PPK, Greither’s disease, Olmsted syndrome and Papillon–Lefevre Syndrome (PLS) should be differentiated from MdM. MdM is characterized by its progrediens and transgrediens clinical manifestations, such as AR PPK. In contrast, the lesions in Nagashima-type PPK are not progressive, and the pathogenic gene is usually to be SERPINB7 [15]. Greither’s disease is similar to MdM in terms of clinical features but differs because it exhibits an autosomal dominant hereditary pattern [16]. Olmsted syndrome is an AD disease affecting the perioral area [17]. PLS is also a rare autosomal recessive disease with typical signs of PPK and severe early-onset periodontitis; onset usually occurs between 1–4 years old [18], and mutations in the cathepsin C gene (*CTSC*) have been reported to be related to the pathogenesis of PLS [19].

Our patients both showed keratoderma on the palms and soles as well as an autosomal recessive hereditary pattern. P2 showed typical diffuse hyperkeratosis progressing with age, whereas P1 had a milder phenotype. The lesions in P1 were restricted to the palms and soles, with no macerations or repeated infections. There may be various reasons for these differences, including the different ages, medical histories, and treatments, among others. However, the symptoms of P1 resembled an MdM variant: PPK of the Gamborg-Nielsen type (PPK-GN). PPK-GN has been classified as an MdM variant due to the presence in the *SLURP1* gene mutations. PPK-GN patients show a less severe phenotype than MdM patients. PPK-GN has been reported in Japanese, Swedish and Chinese populations [15, 20, 21]. Overall, hyperkeratosis of lesions in PPK-GN can be mild, and the nails are not affected. Differentiation between MdM and PPK-GN depends on clinical manifestations and on mild microstructural differences observed under the microscope. Unfortunately, the parents of P1 refused to provide a tissue biopsy. Thus, we cannot definitely classify P1 as having PPK-GN.

The *SLURP1* gene consists of 3 exons and 2 introns. Over 20 mutations in *SLURP1* in MdM have been reported (Additional file 1). Several cases of MdM with *SLURP1* mutations have also been reported in China [22–28], with c.256G > A (p.Gly86Arg) being the pathogenic mutation in these cases. In the present study, we detected for the first time the compound heterozygous mutation c.243C > A (Asp81Glu) and c.256G > A (Gly86Arg). Only four rare types of compound heterozygous mutations have been reported to date [21, 29–31], but none was found in a Chinese individual. In previous studies, 4 patients in 3 Chinese families from mainland China were reported to have MdM due to the same homozygous mutation (c.256G > A). Therefore, c.256G > A might be a mutation hotspot in Chinese patients with MdM, suggesting that it should be a priority for diagnostic gene analysis. According to a previous report, a female with heterozygous missense mutations in exon 3 showed mild clinical manifestations and exhibited mild palmar lesions [32]. Our patient with a compound heterozygous mutation, including c.256G > A, also presented mild symptoms. Whether this is due to the mutation type or the young age still needs investigation. We also detected the homozygous mutation c.211C > T (p.Arg71 > Cys) in P2, who was from a consanguineous Chinese family. Her parents both carried a heterozygous mutation but showed no skin changes. This mutation has been reported once in a Japanese family [33] but is novel in Chinese individuals.

The algorithms used in this study have different strategies to predict the pathogenicity of mutations. MutationTaster evaluates DNA sequences, whereas SIFT, FATHMM and PANTHER focus on evolutionary conservation. PolyPhen-2 is based on comparison of protein 3D structure. PROVEAN is based on protein sequence alignment. In total, nine algorithms were used to analyze mutations in exons, including the novel mutation. Not all algorithms identified D81E as damaging; for example, MutationTaster indicated that it might be a polymorphism. However, this mutation is considered pathogenetic owing to the high conservation in different species (Fig. 3) and the “disease causing” predictions from other algorithms (Table 1). To further investigate the impact of the mutations, we used AlphaFold2 to construct the structures of the proteins and PyMOL for analysis. The comparison showed that changing aspartic acid to glutamic acid reduces hydrogen bonding in the protein, which is essential to maintain protein stability. Although aspartic acid and glutamic acid are both polar negatively charged residues, the substitution results in a decline in hydrogen bonds. Similar observations were made for c.256G > A, c.211C > T and other mutations reported previously. Although AlphaFold2 is proven to be excellent in single-chain protein structure modelling, some researchers claim that it might not be as useful for missense mutation prediction [13, 34]. To test the efficiency of AlphaFold2 on SLURP1, a single-chain protein with 103 amino acids, structure modelling, we examined the precision of AlphaFold2 by Ramachandran plots. The results are satisfying because over 95% of the residues are in the most favored regions and additional allowed regions (Fig. 4b), illustrating that AlphaFold2 is an ideal algorithm for our study.

By reviewing the literature, we found that mutations appear to correlate geographically. For example, the c.211C > T mutation has been detected only in Chinese and Japanese families, whereas the c.256G > A mutation is common in Asia, including in Chinese, Palestinian, Turkish, Korean, Pakistani, and Indonesian patients. Patients from Middle East countries, including Palestine, Algeria and Tunisia, have been reported to share the same c.82delT mutation [9, 35, 36]. This was also reported by Radiono et al. in 2017, whereby c.82delT was the most common mutation in ethnic groups from countries surrounding the Mediterranean Sea and c.43 T > C was common in Europeans [33]. Some might argue that the c.256G > A mutation is not restricted to Asian patients because it has also been detected in ethnic groups from seven countries, including the USA and Australia. However, we found that these patients to actually be Asian immigrants. The close relationship between geography and specific mutations suggests a map of MdM gene mutations.

## Conclusions

In conclusion, we discovered one novel compound heterozygous mutation in a Chinese MdM family. The substitution of aspartic acid with glutamic acid results in hydrogen bond reduction, which might cause the structure of the SLURP1 protein to be unstable. The pathogenicity of this mutation is supported by algorithms. We also detected a previously reported mutation in a Chinese patient for the first time, which expands our knowledge of MdM. We also review the literature on *SLURP1* mutations to further understanding of the molecular mechanism of this disease.

## Supplementary Information

**Additional file 1.** Identification of SLURP1 mutations.

## Data Availability

All the data generated or analyzed during this study are available from the corresponding author by reasonable request. The compound heterozygous mutation c.243C > A and c.256G > A, and a homozygous mutation c.211C > T were submitted to ClinVar database (https://www.ncbi.nlm.nih.gov/clinvar/). The submission number was SCV002584524, SCV003921121 and SCV003930372 respectively.

## Abbreviations

*ARS-B*: Arylsulfatase B
HGMD: Human gene mutation data base
dbSNP: Single nucleotide polymorphism data base
ExAc: Exome Aggregation Consortium
gnomAD: Genome Aggregation Database
MDM: Mal de Meleda
OMIM: Online Mendelian inheritance in man
PPK: Palmoplantar Keratoderma
*SLURP1*: Secreted LY6/PLAUR domain containing 1
