# Supplementary material for: Identification of a novel compound heterozygous mutation and a homozygous mutation of SLURP1 in Chinese families with Mal de Meleda
Source: BMC Med Genomics. 2023 Jul 1;16:152. doi: 10.1186/s12920-023-01580-1 (PMC10314434; doi:10.1186/s12920-023-01580-1)
Supplement: Supplementary file 1 — Additional file 1. Identification of SLURP1 mutations. [file 12920_2023_1580_MOESM1_ESM.docx]

Additional file 1 Identification of *SLURP1* mutations

| Nationality | Mutation | Type | Allele | Site | | | Age | Sex | | Consanguineous | Reference |
| --- | --- | --- | --- | --- | --- | --- | --- | --- | --- | --- | --- |
| Algerian | c.82delT | deletion | Homozygous | | Exon2 | / | | | / | / | J Fischer, 2001 |
| Algerian | c.178+1G>A | splicing | Homozygous | | Intron2 | / | | | / | / | J Fischer, 2001 |
| Chinese M | c.256G>A | missense | Homozygous | | Exon3 | 17 | | | Female | / | Zhou W, 2021 |
| Chinese M | c.256G>A | missense | Homozygous | | Exon3 | 25 | | | Male | / | Jia WX, 2020 |
| Chinese M | c.256G>A | missense | Homozygous | | Exon3 | 48 | | | Male | No | Pan Y, 2017 |
| Chinese M | c.256G>A | missense | Homozygous | | Exon3 | Male (2,4) Female (11) | | | 2Male, 1Female | / | Zhang J,2016 |
| Chinese M | c.211C>T | missense | Homozygous | | Exon3 | 27 | | | Female | YES | This report |
| Chinese M | c.243C>A, c.256G>A | missense | Compound Heterozygous | | Exon3 | 11 | | | Female | No | This report |
| Chinese T | c.256G>A | missense | Homozygous | | Exon3 | 4 | | | Female | No | Kudo M, 2020 |
| Chinese T | c.256G>A | missense | Homozygous | | Exon3 | / | | | 2Male, 2Female | No | Tjiu JW, 2011 |
| Chinese T | c.256G>A | missense | Homozygous | | Exon3 | 22 | | | Female | No | Chao SC, 2006 |
| Dutch | c.43T>C | missense | Homozygous | | Exon1 | 4 | | | / | / | Nellen RG, 2013 |
| Dutch | c.43T>C, c.212G>C | missense | Compound Heterozygous | | Exon1, exon3 | 17 | | | Male | No | Nellen RG, 2009 |
| Bedouin | c.1A>C | missense | Homozygous | | Exon1 | 12 | | | M | Yes | Eckl KM, 2003 |
| German | c.43T>C | missense | Homozygous | | Exon1 | / | | | 2Male, 2Female | No | Eckl KM, 2003 |
| Indian | c.58+5G>T | splicing | Homozygous | | Intron1 | 15 | | | Female | Yes | Nellen RG, 2015 |
| Indonesian | c.256G>A | missense | Homozygous | | Exon3 | 40 | | | Female | No | Taylor JA, 2016 |
| Japanese | c.154A>G (GN) | missense | Homozygous | | Exon2 | 80 | | | Male | Yes | Kunisada M, 2019 |
| Japanese | c.211C>T | missense | Homozygous | | Exon3 | / | | | / | / | Maeda T, 2010 |
| Japanese | c.58+1G>C | splicing | Homozygous | | Intron1 | 56 | | | Male | / | Sakabe J, 2014 |
| Javanese | c.271-273TCTdel | deletion | Homozygous | | Exon3 | Male (14) | | | 1Male, 2Female | No | Radiono S, 2017 |
|  |  |  |  |  |  | Female (22,12) | | |  |  |  |
| Korean | c.256G>A, 286C>T | missense, nonsense | Compound heterozygous | | Exon3 | 15 | | | Female | No | Oh YJ, 2011 |
| Libyan | c.256G>A | missense | Homozygous | | Exon3 | 28 | | | Female | Yes | Bchetnia M, 2015 |
| Pakistani | c.286C>T | missense | Homozygous | | Exon3 | / | | | 5Male, 4female | Yes | Wajid M, 2009 |
| Pakistani | c.256G>A | missense | Homozygous | | Exon3 | / | | | 5Male | Yes | Wajid M, 2009 |
| Pakistani | c.58+1G>A | splicing | Homozygous | | Intron1 | / | | | 2Male,  3Female | Yes | Wajid M, 2009 |
| Pakistani | c.44C>T | nonsense | Homozygous | | Exon1 | Male (16,11), Female (27,22,45,15,13) | | | 2Male, 5Female | No | Akbar A, 2019 |
| Pakistani | c.2T>C | missense | Homozygous | | Exon1 | / | | | 4Male, 1Female | yes | Shah K, 2016 |
| Palestinian | c.82delT | deletion | Homozygous | | Exon2 | Male (46), Female (42) | | | 1Male, 1Female | Yes | Bergqvist C, 2018 |
| Palestinian | c.256G>A | missense | Homozygous | | Exon3 | / | | | 5Male, 8Female | Yes | Eckl KM, 2003 |
| Scottish | c.43T>C, c.82delT | missense, deletion | Compound heterozygous | | Exon1, exon 2 | / | | | / | No | Marrakchi S, 2003 |
| Swedish | c.43T>C | missense | Homozygous | | Exon 1 | / | | | / | / | Zhao L, 2014 |
| Swedish | c.43T>C, c.280T>A | missense | Compound heterozygous | | Exon1, exon 3 | / | | | / | / | Zhao L, 2014 |
| Tunisian | c.82delT | deletion | Homozygous | | Exon2 | 40 | | | Female | Yes | Bchetnia M, 2013 |
| Tunisian | c.82delT | deletion | Homozygous | | Exon2 | 40 | | | Male | No | Bchetnia M, 2013 |
| Tunisian | c.82delT | deletion | Homozygous | | Exon 2 | 70 | | | Male | Yes | Arousse A, 2019 |
| Tunisian | c.296G>A | missense | Homozygous | | Exon3 | / | | | 11Male, 9Female | Yes | Marrakchi S, 2003 |
| Tunisian | c.296G>A | missense | Homozygous | | Exon3 | Male (2), Female (13) | | | 1Male, 1Female | Yes | Bchetnia M, 2013 |
| Tunisian | c.229T>C | missense | Homozygous | | Exon3 | / | | | / | Yes | Charfeddine C, 2003 |
| Tunisian | c.82delT | deletion | Homozygous | | Exon2 | / | | | / | Yes | Charfeddine C, 2003 |
| Tunisian | c.296G>A | missense | Homozygous | | Exon3 | / | | | / | Yes | Charfeddine C, 2003 |
| Turkish | c.287 G>C | missense | Homozygous | | Exon3 | 19 | | | Male | No | Gurel G, 2019 |
| Turkish | c.256G>C | missense | Homozygous | | Exon3 | / | | | 2Female | Yes | Eckl KM, 2003 |
| Turkish | c.129C>A | nonsense | Homozygous | | Exon 2 | 15 | | | Male | Yes | Muslumanoglu MH, 2006 |
| Turkish | c.286C>T | nonsense | Homozygous | | Exon3 | / | | | / | Both | Hu G, 2003 |
| Turkish | c.293T>C | missense | Homozygous | | Exon3 | 8 | | | Female | No | Muslumanoglu MH, 2006 |
| Turkish | c.244C>T | missense | Homozygous | | Exon3 | 27 | | | Male | No | Gruber R, 2011 |

Abbreviation: Chinese M: Chinese mainland; Chinese T: Chinese Taiwan
